# Intrafamilial Phenotype Variability in Two Male Siblings, With X-linked Juvenile Retinoschisis and Dorzolamide Treatment Effect in the Natural History of the Disease

**Published:** 2019

**Authors:** Panayiotis CHRISTODOULOU, George TANTELES, Nayia NIKOLAOU, Ioannis KATSIMBRIS, Maria STEFANIOTOU

**Affiliations:** 1 Ophthalmology Clinic, General Hospital of Patras, Patras, Greece; 2 Clinical Genetics Clinic, Cyprus Institute of Neurology and Genetics, Nicosia, Cyprus; 3 Division of Ophthalmology, University Hospital of Ioannina, Greece.

**Keywords:** X-linked Retinoschisis, Dorzolamide, Central macular thickness, Optical coherence tomography

## Abstract

To investigate how genotype is related to phenotype and document correlations of genotype-phenotype with response of topical administration of dorzolamide in siblings affected with X-linked juvenile retinoschisis (XLRS). We performed a retrospective study on two male siblings (four eyes) with XLRS, who were treated with topical installation of dorzolamide. Clinical diagnosis was supported with familial genetic analysis with bi-directional Sanger sequencing of RS1 pathogenic variant. Optical coherence tomography (OCT), fundus fluorescein angiography (FFA), ultrasound scan (U/S) and electroretinogram (ERG) were used in the evaluation. Central macular thickness (CMT) and best corrected visual acuity (BCVA) were recorded monthly for eighteen months. We performed genetic analysis in their family for mutations in the gene that encodes the protein retinoschisin, responsible for retinoschisis (RS1). It was proved that phenotype variability might be related to the same pathogenic variant. While there was an improvement in BCVA and OCT central macular thickness in the patient with the mild form of disease, the visual acuity and the OCT scans of the patient with severe form of disease did not improve. Intrafamilial phenotypic variability between individuals sharing identical pathogenic variant was documented. Both our patients had a pathogenic variant in a hemizygous state at a genomic location in exon 6 of the RS1 gene; Frameshift mutation that is likely to cause protein truncation was identified which is suggested to result in greater clinical severity. Consequently, it was found that response to dorzolamide is correlated to phenotypic severity.

## INTRODUCTION

X-linked juvenile retinoschisis (XLRS) is a quite rare disorder with a prevalence ranging between 1:5000 to 1:25000 [1]. It is caused by pathogenic mutations in *RS1*, a gene on Xp22.13 coding retinoschisin [2]. It represents the most common cause of juvenile macular degeneration in males and characterized by degenerated vitreous and retinal separation between the nerve fiber and ganglion cell layer [3]. In most cases, carrier females have unremarkable visual function and electroretinography (ERG) [4]. Both inter- and intrafamilial variability in phenotypic expression have been reported [5]. Affected males typically have stellate-like cystic maculopathy or foveal schisis which is predominately inferotemporal in 50% of individuals [6]. Deterioration in visual acuity is typically evident in affected males, between the ages of five and 10 years. Progressive visual deterioration frequently occurs after the fourth decade of life, before which visual impairment is usually mild [7]. During the later stages, the disease may be complicated with vitreous hemorrhage, choroidal sclerosis and/or retinal detachment as a result of low adhesiveness. Retinal atrophy resulting in blindness may rarely implicate the disease. Characteristic features detected by ERG include predominant or selective b-wave amplitude reduction with relative preservation of the a-wave amplitude in affected males [6]; however, recent studies have shown variability [7]. Individuals can have a technically normal ERG in which the b-wave is still present [8]. Therefore, a normal ERG cannot exclude the diagnosis. Optical coherence tomography (OCT) is used to analyze retinal structures in affected males to characterize forms of deeper retinal pathology [9]. The typical cystic stellate maculopathy of XLRS involves the deeper retinal layers [10].

Topical use of carbonic anhydrase inhibitors has been used to reduce intraocular pressure. It has been described to be effective for the treatment of cystoid macular edema in selected patients with retinitis pigmentosa [11] and various groups investigated the effect of topical dorzolamide for the treatment of similar cystic lesions at the fovea in patients with XLRS [12]. We here presented two Greek male siblings with molecularly confirmed XLRS who underwent fundus photography, OCT, fluoroangiography, ERG, visual field test and ultrasonography. Optical coherence tomography before and after the treatment with potent topical carbonic anhydrase inhibitor, dorzolamide, was used to monitor the disease.

## METHODS

**Figure 1:**
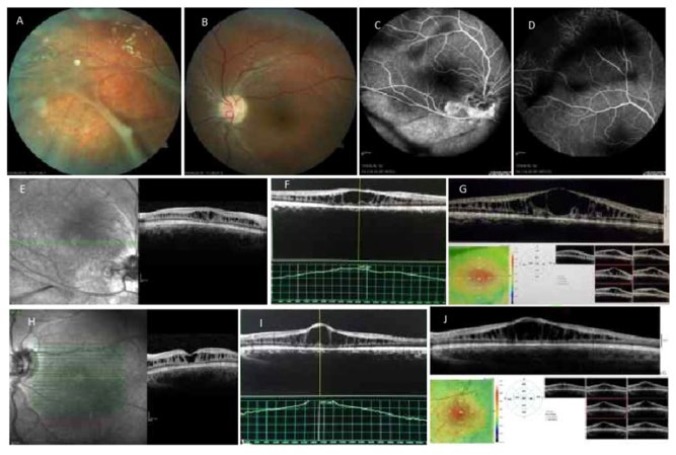
Patient 1. Fundus Photos Showing Stellate Maculopathy and Vitreous Veils (A, B). Fundus Fluorescein Angiography Showed Veils With no Macular Leaking (C, D). Optical coherence tomography Images at First Visit (OD:E, OS:H) Eight (OD:F. OS:I ) and Eighteen Months Following Commencement of Treatment (OD:G, OS:J). Right eye (OD); Left eye (OS)

A 15-year-old boy (patient 1) was admitted for consultation to the Vitreoretinal Clinic of the General Hospital of Patras due to uncorrectable bilateral visual acuity reduction. Patient had no ocular record of inflammation, trauma or infection. Best corrected visual acuity (BCVA) in both eyes (OU) was 2/10. Ocular mobility and anterior segment biomicroscopy had normal findings. Applanation tonometry recorded 12 millimeter of mercury (mmHg) in both eyes. Indirect ophthalmoscopic fundus examination showed indicative radiating cystoid spaces, also established as spoke wheel-like maculopathy and peripheral schisis with congenital vascular veils. Peripheral dentritiform lesions consisting of occluded and sheathed vessels. Findings were more extreme in the right eye (Fig 1 A&B). Investigation with ultrasonography had normal findings. Fluorescein angiography showed window defects, but no leakage of dye in the cystoids spaces in both cases (Fig 1 C&D). The OCT (Heidelberg Spectralis) results revealed retinal cleavage into two distinct planes superficially connected with vertical palisades separated by low reflective spaces most conspicuous in the foveal region in the right eye (OD) at first visit (Fig. OD:E, Left eye (OS):H) eight (Fig 1 OD:F. OS:I) and eighteen months following commencement of treatment (Fig1 OD:G, OS:J).

**Figure 2:**
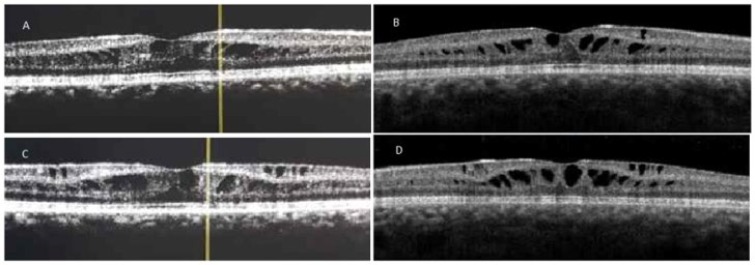
Patient 2. (A) Optical coherence tomography Images at First Visit (OD:A, OS:C) and Eighteen Months following Commencement of Treatment (OD:B, OS:D), Central macular thickness Was Significantly Reduced (OU). Right eye (OD); Left eye (OS); both eyes (OU).

Electroretinogram was recorded according to the International Society for Clinical Electrophysiology of Vision (ISCEV) protocol [8], retinal electric activity was reduced in both eyes with maximum response of the OD: 102,6 microwatt (μV) /41,5 millisecond (ms) and of the OS: 137,5 μV/42,0 ms. Visual fields 24-2 test showed mean deviation -23,52 decibel (dB) and -22,66dB of the right and left eyes, respectively. Their family medical and ophthalmic history was non-contributory. Elder brother’s (Patient 2) ophthalmic examination showed BCVA OU: 7/10, biomicroscopy revealed foveal stellate pattern, OCT examination confirmed the cystic macular changes, visual fields test showed a mild reduction of indices Mean Deviation (MD): OD-3.29, OS-3.09. Both patients received topically 2% dorzolamide three times daily. Both patients were re-evaluated every month. Repeated BCVA and OCT measurements showed no significant improvement either in visual acuity or in retinal anatomy in the first case. However, in the second case there was a significant improvement in the visual acuity in both eyes to 8/10. Cystic foveal lesions and central macular thickness were reduced, (OD from 372 micrometer [μm] to 316μm and OS from 360μm to 327μm), in both eyes (Fig 2).

**Figure 3:**
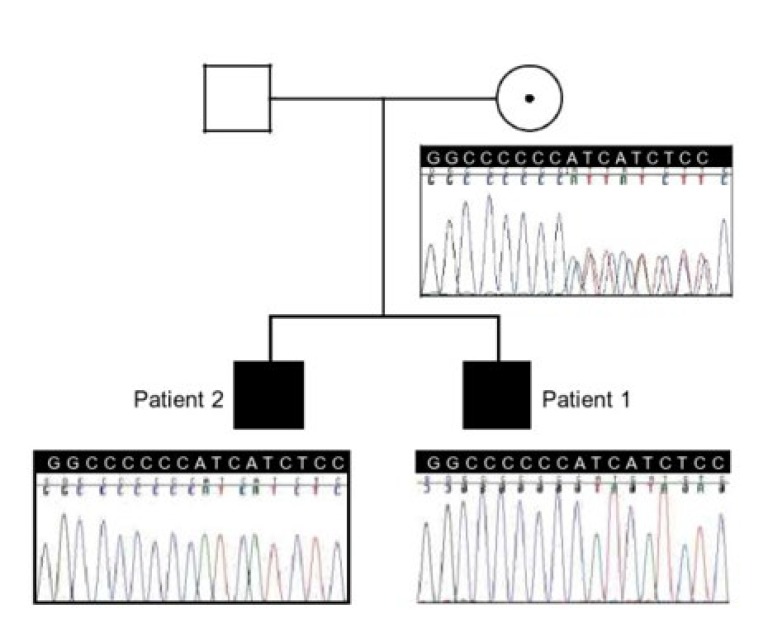
Pedigree Showing Two Affected Male Siblings and Their Mother, Who Is a Healthy Carrier. The Corresponding Chromatograms Show the c.578_579insC (p.His194fsX263) Variant in Exon 6 of RS1; a gene on Xp22.13 coding retinoschisin.

Bi-directional Sanger sequencing of *RS1* revealed a pathogenic variant (c.578_579incC;p.HisfsX263) in a hemizygous state (genomic location X:18660220-18660221) in exon 6 of this gene within a stretch of six cytosine nucleotides (c.574-579) which represents a frameshift mutation likely to cause protein truncation. Pathogenic variants have been identified in this cytosine stretch in multiple families with retinoschisis, which represents a mutational hotspot [1]. Complex segregation analysis determined that a mutation was present in a hemizygous state in his brother and his healthy mother as well (Fig 3).

The study was conducted in accordance with the ethical standards declared in the declaration of Helsinki and was approved by an institutional review board; a written informed consent was obtained from both patients. Data was collected to note Ethics Committee approval.

## Discussion

X-linked juvenile retinoschisis is characterized by decreasing visual acuity until the fifth or sixth decades of life when macular atrophy happens. Averagely, visual acuity is around 3/10 at age 20, gradually declining to 1/20 by age 60, usually because of macular changes [13]. During the first two decades of life visual acuity is around 3/10, gradually reducing to 1/10 by age 60, usually due to macular atrophy. In our report patients were under 20 years, but patient number 2 BCVA was much higher (8/10) than patient number 1 (2/10) which indicates potential difference in visual acutity due to phenotype variability. Apposition of the retinal layers due to coalescent cysts primarily in the outer plexiform and adjacent nuclear layers results in retinoschisis cavity. Foveal schisis occurs in 98% to 100% of patients [8]; the inferotemporal quadrant is the principal site of lesion location [8]. *RS1 *codes for retinoschisin and is the only gene known to cause XLRS. Studies on gene expression and immunolocalization of the typical protein product signify that it is expressed within the photoreceptors and has a complex interaction within retina cells. Retinoschisin is most highly expressed in the inner segments of the photoreceptors in human eye sections and other mammals. It is a 224-amino acid secretory protein (NP_000321.1) that exists as a novel disulfide-linked octamer [14]. Our study revealed a pathogenic variant (c.578_579incC; p.HisfsX263) in a hemizygous state (genomic location X: 18660220-18660221) in exon 6 of this gene within a stretch of six cytosine nucleotides which represents a frameshift mutation likely to cause protein truncation.

Pathogenic variants are predominantly missense and clustered in exons 4–6, encoding the discoidin domain, although splice site, insertions, deletions mutations have been described. It has been reported that missense mutations lead to disease pathology by at least one of the following three mechanisms [14]; interfering with retinoschisisin secretion, octamerisation or allowing secretion and octamerisation but interfere with protein function. In our analysis the pathogenic variant was also located in exon 6 of the gene whithin a stretch of six cytosine nucleotides (c.574-579) representing a frameshift mutation which interferes with protein function, a molecule responsible to maintain adhesion of the structural integrity of the photoreceptor-bipolar synapse. Previous studies reported [12, 13, 15] efficacy of topical carbonic anhydrase inhibitors for the treatment of stellate maculopathy in XLRS without correlating the response to pathogenic variant and phenotype as represented by layers of disease severity. Significant correlations of genotype-phenotype in XLRS is a subject under debate. Certain studies state that variants that putatively cause protein truncation result in higher clinical severity [8]. In other studies it was stated that variants involved in milder phenotypic abnormalities are often seen in patients with missense variants [16]. In our investigation, treatment efficacy was not unequivocal or independent of genotype and phenotype and that mild disease can also result from variants involved in higher clinical severity as those causing protein truncation. Consequently, response to dorzolamide is correlated to phenotypic severity. Whether this is due to residual RS1 gene expression or unidentified parameters remains to be further investigated.

## CONCLUSION

Intrafamilial phenotypic variability between individuals with XLRS sharing identical pathogenic variant was documented. Both our patients had a pathogenic variant in a hemizygous state at a genomic location in exon 6 of the *RS1 *gene; Frameshift mutation that is likely to cause protein truncation was identified which is suggested to result in greater clinical severity; contrary to our cases where both extremes of the severity spectrum were identified. Consequently, response to dorzolamide is correlated to phenotypic severity.
